# Postconditioning in major vascular surgery: prevention of renal failure

**DOI:** 10.1186/s12967-014-0379-7

**Published:** 2015-01-27

**Authors:** Peter Aranyi, Zsolt Turoczi, David Garbaisz, Gabor Lotz, Janos Geleji, Viktor Hegedus, Zoltan Rakonczay, Zsolt Balla, Laszlo Harsanyi, Attila Szijarto

**Affiliations:** 1st Department of Surgery, Semmelweis University, Budapest, Hungary; 2nd Department of Pathology, Semmelweis University, Budapest, Hungary; Eötvös Loránd University, Faculty of Science, Institute of Mathematics, Budapest, Hungary; First Department of Internal Medicine, University of Szeged, Szeged, Hungary

**Keywords:** Myoglobinuria, Postconditioning, Renal microcirculation, HSP72

## Abstract

**Background:**

Postconditioning is a novel reperfusion technique to reduce ischemia-reperfusion injuries. The aim of the study was to investigate this method in an animal model of lower limb revascularization for purpose of preventing postoperative renal failure.

**Methods:**

Bilateral lower limb ischemia was induced in male Wistar rats for 3 hours by infrarenal aorta clamping under narcosis. Revascularization was allowed by declamping the aorta. Postconditioning (additional 10 sec reocclusion, 10 sec reperfusion in 6 cycles) was induced at the onset of revascularization. Myocyte injury and renal function changes were assessed 4, 24 and 72 hours postoperatively. Hemodynamic monitoring was performed by invasive arterial blood pressure registering and a kidney surface laser Doppler flowmeter.

**Results:**

Muscle viability studies showed no significant improvement with the use of postconditioning in terms of ischemic rhabdomyolysis (4 h: ischemia-reperfusion (IR) group: 42.93 ± 19.20% vs. postconditioned (PostC) group: 43.27 ± 27.13%). At the same time, renal functional laboratory tests and kidney myoglobin immunohistochemistry demonstrated significantly less expressed kidney injury in postconditioned animals (renal failure index: 4 h: IR: 2.37 ± 1.43 mM vs. PostC: 0.92 ± 0.32 mM; 24 h: IR: 1.53 ± 0.45 mM vs. PostC: 0.77 ± 0.34 mM; 72 h: IR: 1.51 ± 0.36 mM vs. PostC: 0.43 ± 0.28 mM), while systemic hemodynamics and kidney microcirculation significantly improved (calculated reperfusion area: IR: 82.31 ± 12.23% vs. PostC: 99.01 ± 2.76%), and arterial blood gas analysis showed a lesser extent systemic acidic load after revascularization (a defined relative base excess parameter: 1^st^ s: IR: 2.25 ± 1.14 vs. PostC: 1.80 ± 0.66; 2^nd^ s: IR: 2.14 ± 1.44 vs. PostC: 2.44 ± 1.14, 3^rd^ s: IR: 3.99 ± 3.09 vs. PostC: 2.07 ± 0.82; 4^th^ s: IR: 3.28 ± 0.32 vs. PostC: 2.05 ± 0.56).

**Conclusions:**

The results suggest a protective role for postconditioning in major vascular surgeries against renal complications through a possible alternative release of nephrotoxic agents and exerting a positive effect on hemodynamic stability.

## Background

An acute deterioration of renal function is a common observation after major vascular surgery [1]. The etiology and mechanisms of the perioperative renal dysfunction can be related to preexistent functional compromise, perioperative systemic or local hemodynamic disturbances, injury to the urinary tract, pre- and perioperative drug-induced nephropathy, neuroendocrine stress response and inflammatory changes, and renal ischemia due to an emerging suprarenal aortic clamping [2,3]. Another explanation for the pathogenesis is the muscular injury of the lower limb [4,5]. Crossclamping of the aorta or major arteries leads to an ischemia-reperfusion (IR) injury to a large skeletal muscle mass. Myocyte damage causes a release of intracellular nephrotoxic contents (like excess myoglobin) into the circulation, triggers local inflammatory processes, and can lead to a profound metabolic disorder with hyperkalemia, hypocalcemia, and systemic acidosis [6].

Several brief episodes of ischemia and reperfusion induced immediately after the relief of a longer occlusion - a method called postconditioning - has been widely studied in recent years and claimed to exert protective effect against the IR injury [7]. McAllister et al. were the first who studied postconditioning in a skeletal muscle model and demonstrated a reduction in muscle infarction size, also in muscle myeloperoxidase activity and mitochondrial calcium concentration in a porcine muscle flap model [8]. In a former study of our scientific group, the postoperative systemic complications, the so-called reperfusion syndrome was also investigated in a rat abdominal aorta occlusion model, and we demonstrated a significant improvement of postoperative systemic inflammatory response and redox homeostasis [9].

This present study further investigates the effect of postconditioning on muscle injury and the evolving renal dysfunction as a serious postoperative complication in a rat model of open abdominal aortic surgery.

## Materials and methods

### Surgical preparation

Inbred male Wistar rats (250-280 g, Charles River Hungary Ltd, Budapest, Hungary) were anesthetized (intraperitoneal injection of 75 mg/kg ketamine, and 7.5 mg/kg xylazine). In the 4 h reperfusion subgroups, a 22-gauge catheter was inserted into the right carotid artery to monitor arterial blood pressure.

The right jugular vein was cannulated for administration of intraoperative saline infusion (3 ml/kg/h), maintaining anesthesia (25 mg/kg/h ketamine, 2.5 mg/kg/h xylazine), and injection of heparin (60 IU/kg before the aortic clamping).

Through a median laparotomy the retroperitoneal space was opened. Except for the sham-operated animals, all rats underwent 180 min of bilateral lower limb ischemia by infrarenal crossclamping of the abdominal aorta using an atraumatic microvascular clip (Aesculap FT260T; B.Braun AG, Melsungen, Germany).

The experiment was approved by the Animal Care Committee of Semmelweis University (License No: 22.1/2409/3/2011) in consistence with the US National Institute of Health guidelines (Publication No. 85–23, revised 1996; MD, USA).

### Experimental groups

1. IR (ischemia-reperfusion) group (n = 24): After 180 min lower limb ischemia, a reperfusion period of 240 min followed (n = 8). The rest of the animals were allowed a survival time after revascularization. Sample-taking was performed 24 h (n = 8) and 72 h (n = 8) later.
2. The Postconditioned (PostC) group (n = 24) was subjected to IR and postconditioning, consisting of 6 cycles of 10-seconds’ aortic declamping and 10 seconds’ reocclusion at the onset of reperfusion. (Reperfusion intervals: 4, 24, and 72 h, 8 animals in each subgroup).
3. In the sham-operated group (n = 15), rats were not subjected to aortic clamping, other interventions and measurements were performed in the same manner as in the animals subjected to limb ischemia. Samples were taken 4 (n = 5), 24 (n = 5) and 72 h (n = 5) postoperatively.
4. For base excess measurements, additional 10 animals underwent 180 min of bilateral lower limb ischemia. In 5 of the 10 animals postconditioning was performed. 0.3 ml blood samples were taken from the right common carotid artery 5 times in each animal: just before the onset of reperfusion and at the end of the 1^st^, 2^nd^, 3^rd^, 4^th^ min of the reperfusion.

### Laboratory measurements

Blood samples were harvested from the right ventricle of the heart by direct puncture.

Urine samples were collected in the 4 h subgroups from the second hour on after declamping (i.e. for 3 h). In the surviving animals urine samples were taken through a laparatomy by a needle puncture of the urinary bladder.

Samples were snap-frozen in liquid nitrogen and stored at −70°C. Analysis was performed by an automated clinical chemistry analyzer (Beckman Coulter AU480/2011, Beckman Coulter Inc, Brea, CA, USA).

### Muscle viability

Rectus femoris muscle samples were rapidly frozen in liquid nitrogen and stored at −70°C until processed. Slices (3 μm) were incubated in dark for 30 min in 0.025% nitroblue tetrazolium chloride (NBT, Sigma-Aldrich Inc, St. Louis, MO, USA), dissolved in pH7.6 tri-hydroxymethyl-aminomethane (TRIS) buffer containing 0.05 mmol/l nicotinamide-adenine-dinucleotide (NADH). Unbound NBT was eliminated from the sections with 30, 60 and 90% acetone solutions in increasing and decreasing concentrations.

Under standardized conditions, photographs were taken, under light microscope (5 randomly selected areas per each animal). Staining density was measured using a morphometric software (Leica Qwin Pro, Leica Microsystems GmbH, Weltzer, Germany). The relative area of a preset wavelength-range (blue: 175/66, green: 105/0, red: 150/0 on the RGB scale) was determined as a quotient of the occupied area and the total area of the picture.

Untreated controls (n = 5) were also involved in the experiment. In all operated animals, viability was expressed as a percentage of the untreated animals’ staining density.

### Anti-myoglobin immunohistochemistry

Kidney samples were fixed in 4% neutral-buffered formalin, washed with phosphate-buffered saline. Endogenous peroxidase activity was blocked with 3% hydrogen-peroxide. Antigen retrieval was performed in 10 mmol/l citrate buffer, in a pH 6.0 medium. For inhibition of nonspecific binding, tissue was blocked with bovine serum albumin. Samples were incubated (12 h, 4°C) with polyclonal anti-human anti-myoglobin antibodies raised in rabbits (1:50, Diagnostic BioSystems Inc, Pleasanton, CA, USA) and hybridized for 1 h with peroxidase-conjugated secondary antibody (EnVision®, Dako Denmark A/S, Glostrup, Denmark). For visualization 3-diaminobenzidine was applied.

### Acid–base status

Arterial blood samples were analysed using Radiometer ABL80 Astrup machine (Radiometer Medical ApS Åkandevej 21 DK-2700, Brønshøj, Denmark). For further calculations, base excess (BE) was chosen as being an indicator of an acidic load of the systemic circulation. For better graphical understanding and comparison of the individual animals, a relative base excess (RBE) parameter was defined: a quotient of each measured BE and the BE registered before the onset of reperfusion.

### Hemodynamics

#### Recording and mathematical processing of hemodynamic parameters

By invasive blood pressure monitoring system (Kent Scientific Corporation, Torrington, CT, USA), blood pressure was recorded by DasyLab V9.00.02. (National Instruments Corporation, Austin, TX, USA), calculating and registering heart rate (HR), systolic (SBP) and diastolic blood pressure (DBP) values every 5 seconds.

Parameters underwent mathematical transformations using c++ (ISO/IEC14882 standard) code edited in Code::Blocks (10.05 rev6283m Code::Blocks Team, USA) and compiled by MinGW (2012., mingw.org). For eliminating the incidental measurement errors and failures due to external influences, some criteria were defined and enforced by deleting those data that failed these preset conditions. The invalidated data were replaced by the last known accepted values. As criteria, upper and lower bounds for SBP, DBP and HR values were adjusted for each individual animal. Sudden changes in these parameters were also considered as results of external factors. Thus data were cancelled and replaced when exceeding a preset percentage of difference compared to the last accepted value. When replacing multiple consecutive faulty values, the percentage interval of admissible parameters was increased exponentially with respect to the duration of the measurement error. The resulting sequences of data underwent further mathematical transformations (Gaussian smoothing) for the purpose of calculations and better comparability of the individual animals. For each parameter a mean of nearby values weighted with Gaussian function was taken.

#### Data analysis

The following calculations were performed on each animal’s blood pressure and heart rate curves:

- (a) Similarly to ’shock index’ , at each time point the quotient of HR and SBP was registered and the mean was taken for the period of ischemia and for reperfusion. Dividing this mean of reperfusion by the mean of ischemia, a quantity was achieved that is characteristic for the hemodynamic changes occurring after revascularization and the efficacy of the given animal’s compensative mechanisms.
- (b) To quantify the observed drop of blood pressure at revascularization, the lowest mean arterial pressure (MAP) was identified within the first 20 min of the reperfusion period. The time intervals were determined between this time point and the beginning of reperfusion, as well as the degree of this drop expressed as percentage of the last registered MAP of the ischemic period.
- (c) After the drop of blood pressure, a gradual rise was apparent in each animal reaching again a plateau state. With help of ’IF function’ of Microsoft Excel (Microsoft Corporation, Redmond, WA, USA), the point in time was determined when mean arterial blood pressure reaches the beginning of the plateau phase.

### Kidney cortical microcirculation

A laser Doppler (DRT4 device; DP1T surface probe, Moor Instruments Ltd, London, UK) surface probe was placed at an identical spot directly on the anterior surface of the left kidney through the incision of laparotomy. Measurements were taken and registered throughout the ischemic period and the 4 h of reperfusion.

Two parameters were calculated: reperfusion area (RA, integral of the reperfusion segment of the graphs, proportional to the average blood-flow during reperfusion), and the plateau maximum (PM, mean of the last, plateau-shaped 10 min of the reperfusion-slope) [10].

For accurate comparison of the curves, each measured flux was calculated as a percentage of baseline flux before the aortic clamping.

### Heat shock protein 72 (HSP72) analysis

Kidney samples in the non-surviving animal groups were homogenized in a buffer medium (10 mmol/l 4-(2-hydroxyethyl)-1-piperazineethanesulfonic acid (HEPES) pH = 7.9, 1.5 mmol/l MgCl_2_, 10 mmol/l KCl, 1 mmol/l dithiothreitol (DTT), 1 mmol/l phenylmethylsulfonyl fluoride (PMSF), 4 mmol/l benzamidine, 100 U/ml aprotinin, Gedeon Richter PLC, Budapest, Hungary) with a sonicator (Cole-Parmer Instrument Co., Chicago, IL, USA). HSP72 determination was performed as described previously with Western blot analysis [11], using rabbit anti-HSP72 antibodies (1:10,000) incubated for 1 h at room temperature and hybridized with horseradish peroxidase-coupled goat anti-rabbit secondary antibody (1:10,000, 1 h; Dako A/S, Glostrup, Denmark) [12]. Bands were visualized by chemiluminescence, scanned and quantified by ImageJ software (NIH, Bethesda, MD, USA). To control for equal loading of protein, the expression of the housekeeping protein glyceraldehyde 3-phosphate dehydrogenase (GAPDH) was also checked on the same membrane by subsequent hybridization with mouse anti-GAPDH (1:10,000 dilution for 1 h; Biodesign International, Saco, ME, USA) and horseradish peroxidase-coupled goat anti-mouse (1:10,000 dilution for 1 h; Dako A/S, Glostrup, Denmark) antibodies.

### Lipid peroxidation

After 4 h of reperfusion, right kidney samples were homogenized, diene conjugate contents were extracted by adding isooctane (5 ml isooctane pro 1 g wet tissue sample). After storing under hypoxic conditions (room temperature, 20 h), the conjugated diene concentrations were detected using a Lumat LB 9051 spectrophoto-meter (Lumat, Berthold, Windbad, Germany) on 232 nm wavelength (AOAC Official Methods of Analysis 1984; 28054 B. 14th edition, Arlington, USA). Formation of diene conjugates represents the first step of lipid peroxidation thus indicates oxidative injury in kidneys.

### Statistical analysis

Data were analysed using IBM SPSS Statistics Version 20 (IBM Corporation, Armonk, NY, USA). Values are presented as means ± standard deviation. For every group of animals the normality of each measured parameter’s distribution was tested using Kolmogorov-Smirnov statistic. Levene’s tests for homogeneity of variances were performed. In cases when homogeneity of variances was assumed, one way ANOVA (analysis of variance) tests were calculated with Scheffe’s post hoc test for between groups comparisons. When heterogenity of variances was assumed, Brown-Forsythe test was performed with Games-Howell post hoc multiple comparisons. RBE measurements were tested using one-way repeated measures ANOVA. GraphPad (GraphPad Software Inc, La Jolla, CA, USA) was used to generate the flow graphs. Any differences were considered statistically significant with p values less than 0.05.

## Results

### Biochemical assessment of the lower limb IR-injury

Elevated serum creatine kinase, aspartate transaminase and lactate dehydrogenase activities were found in both the IR and PostC groups, compared to the sham-operated animals. Until the end of the 3^rd^ postoperative day, necroenzyme levels returned to the normal range. The difference between the IR and the PostC groups proved non-significant at either of the measured time-points (Table 1).

**Table 1:** ***Laboratory measurements for assessment of muscle ischemia***-***reperfusion injury***

	**Creatine kinase**			**Aspartate aminotransferase**			**Lactate dehydrogenase**		
	**Sham**	**IR**	**PostC**	**Sham**	**IR**	**PostC**	**Sham**	**IR**	**PostC**
4 h	257.00 ± 56.32	875.22 ± 206.52	921.00 ± 329.53	162.00 ± 27.50	249.33 ± 78.43	167.00 ± 81.90	256.67 ± 64.49	1075.00 ± 144.33	917.63 ± 217.82
24 h	318.50 ± 28.99	340.25 ± 232.25	285.71 ± 116.79	104.50 ± 51.62	186.63 ± 139.24	239.29 ± 155.17	287.00 ± 21.21	439.75 ± 209.19	423.67 ± 180.20
72 h	197.00 ± 24.04	328.30 ± 138.82	390.43 ± 95.71	56.33 ± 6.66	93.75 ± 83.99	90.50 ± 90.43	217.00 ± 127.28	284.91 ± 186.59	284.88 ± 106.03

Serum necroenzyme levels are expressed in IU/l. Values are given in each subgroup as means ± SD.

### Muscle viability

**Figure 1:**
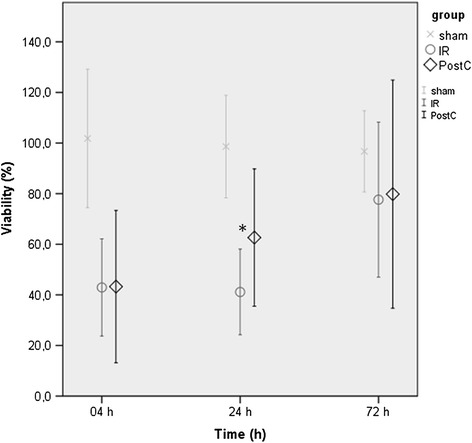
***Muscle viability*** **.** 4 h after revascularization a marked decrease in viabilty was detected in both the IR (ischemia-reperfusion, ○, n = 8) and PostC (postconditioned, ◊, n = 8) groups compared to the sham-operated (x, n = 5) group. On the first postoperative day, a regeneration seems to be in process in the PostC group, while viability remained on a similar level in the IR group (significant difference). 72 h after the revascularization a further improvement can be detected in both subgroups, with no significant difference between either of the subgroups. (Values are expressed as means ± SD, *indicates p < 0.05.).

4 hours after revascularization a marked muscle injury was detected in both ischemic subgroups, without any significant difference. Till the end of the first postoperative day, a regeneration seemed to be in process in the PostC group, while in the IR group viability remained on a similar level as it was 4 hours after reperfusion (significant difference). 72 hours after revascularization, a further improvement could be detected in both subgroups, with no significant difference between the postconditioned and the non-conditioned (IR) animals (Figure 1).

### Anti-myoglobin immunohistochemistry

**Figure 2:**
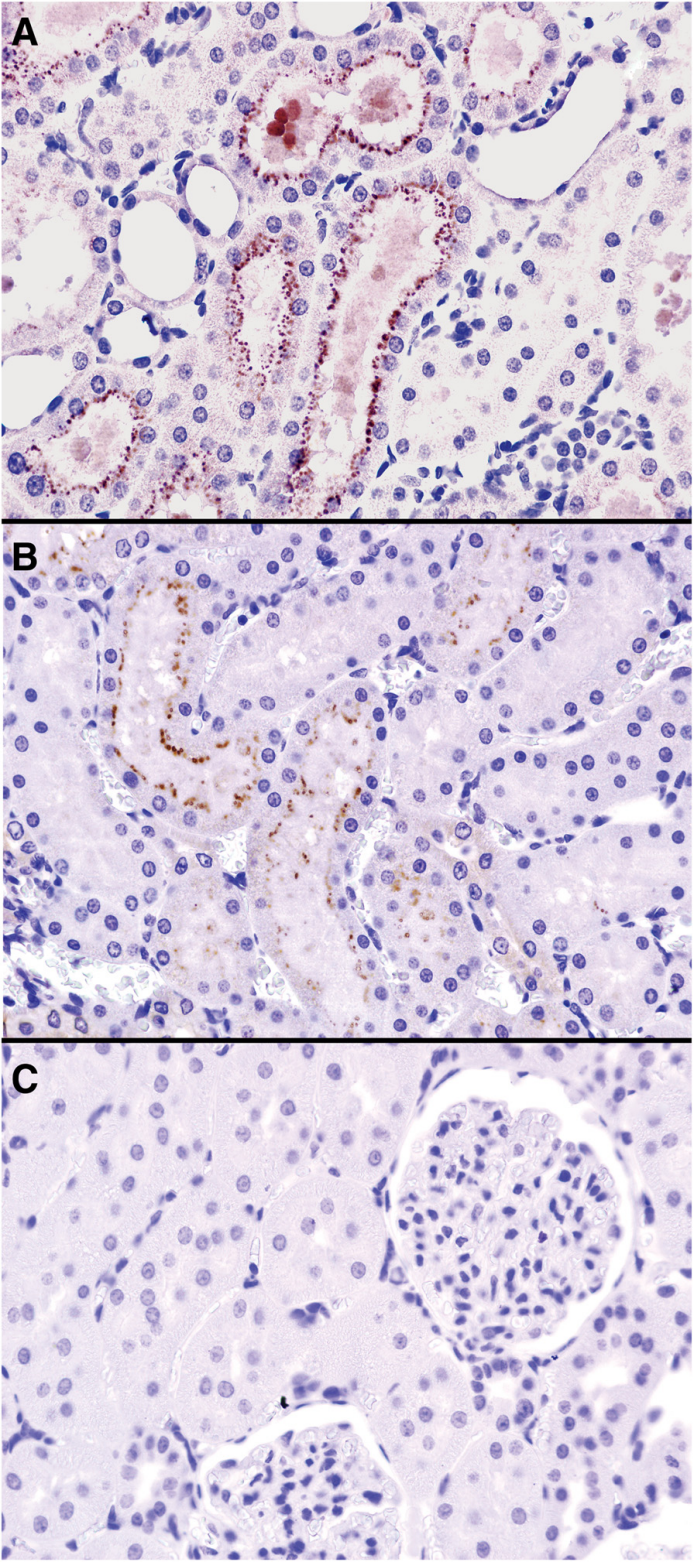
***Anti***-***myoglobin immunohistochemistry*** **(Gill’** **s hematoxylin as background staining**, **200x and 600x magnification).** ***A***: Kidney section of an IR (ischemia-reperfusion) animal (4 h after revascularization) shows myoglobin casts in the lumen of the tubuli, anti-myoglobin-positive vacuoli within the proximal tubular cells. All other stain was considered non-specific binding and negative for the presence of myoglobin. ***B***: Slide of a PostC (postconditioned) animal 4 h after revascularization. Less anti-myoglobin positivity can be detected, ***C***: IR animal 24 h after revascularization, myoglobin epitopes were absent.

4 hours after reperfusion presence of myoglobin epitopes was detected in the lumen of the kidney tubules and also in the apical lysosome vacuoli of the proximal tubular cells. Sections demonstrated a milder myoglobin-load in the PostC animals with less tubular myoglobin casts and sparse presence in the intracellular lysosomes (Figure 2).

24 and 72 hours after revascularization, all kidney sections proved myoglobin-negative (no intraluminal stained casts or intracellular signs, due to probably the dissolved or transformed myoglobin-epitopes).

### Renal function

All animals subjected to limb IR developed acute kidney injury of some extent. For characterization of the evolving renal dysfunction, clinical renal functional parameters were calculated. At the end of the fourth hour of reperfusion, both serum carbamide/creatinine ratio and the fractional sodium excretion (FENa: Clearance_Na_/Clearance_creatinine_*100) indicated a tubular type of kidney failure, but only in the IR group, whereas the values in the PostC group were not characteristic for any type of kidney failure, according to usual definitions. Renal failure index (RFI = [Na]_urine_*[creatinine]_urine_/[creatinine]_plasma_) indicated persistent kidney damage with significantly lower values in the PostC subgroups (Table 2).

**Table 2:** ***Laboratory measurements and calculated parameters for kidney function***

		***4 h***	***24 h***	***72 h***
**Creatinine** [**μmol**/**l**]	*Sham*	67.35 ± 16.27	20.40 ± 7.50	20.35 ± 4.74
	*IR*	219.09 ± 81.92	39.22 ± 3.32	38.07 ± 2.07
	*PostC*	96.25 ± 28.07‡	26.43 ± 6.88^†^	31.10 ± 6.79
**Carbamide** [**mmol**/**l**]	*Sham*	6.31 ± 0.71	5.36 ± 0.49	5.09 ± 0.13
	*IR*	8.03 ± 0.90	6.15 ± 0.29	6.30 ± 0.64
	*PostC*	6.38 ± 0.50^†^	5.55 ± 0.07	5.32 ± 0.29†
**Carbamide/** **Creatinine**	*Sham*	97.54 ± 32.77	276.79 ± 77.78	253.51 ± 60.76
	*IR*	39.90 ± 14.65	159.06 ± 25.14	134.50 ± 32.96
	*PostC*	68.37 ± 24.51^†^	258.40 ± 53.02^†^	199.69 ± 39.40
**FENa** [%]	*Sham*	0.24 ± 0.26	0.34 ± 0.09	0.87 ± 0.12
	*IR*	2.19 ± 1.31	0.94 ± 0.29	0.98 ± 0.25
	*PostC*	0.75 ± 0.50	0.50 ± 0.22	0.26 ± 0.16^†^
**RFI** [**mmol**/**l**]	*Sham*	0.42 ± 0.37	0.49 ± 0.12	0.77 ± 0.56
	*IR*	2.37 ± 1.43	1.53 ± 0.45	1.51 ± 0.36
	*PostC*	0.92 ± 0.32	0.77 ± 0.34^†^	0.43 ± 0.28^†^

Creatinine and carbamide concentrations are measured in serum samples. Values are given in each subgroup as means ± SD. Abbreviations: FENa: fractional sodium excretion. RFI: renal failure index.

^†^p < 0.05 vs. corresponding IR; ‡ p < 0.001 vs. corresponding IR.

### Systemic acid–base status

**Figure 3:**
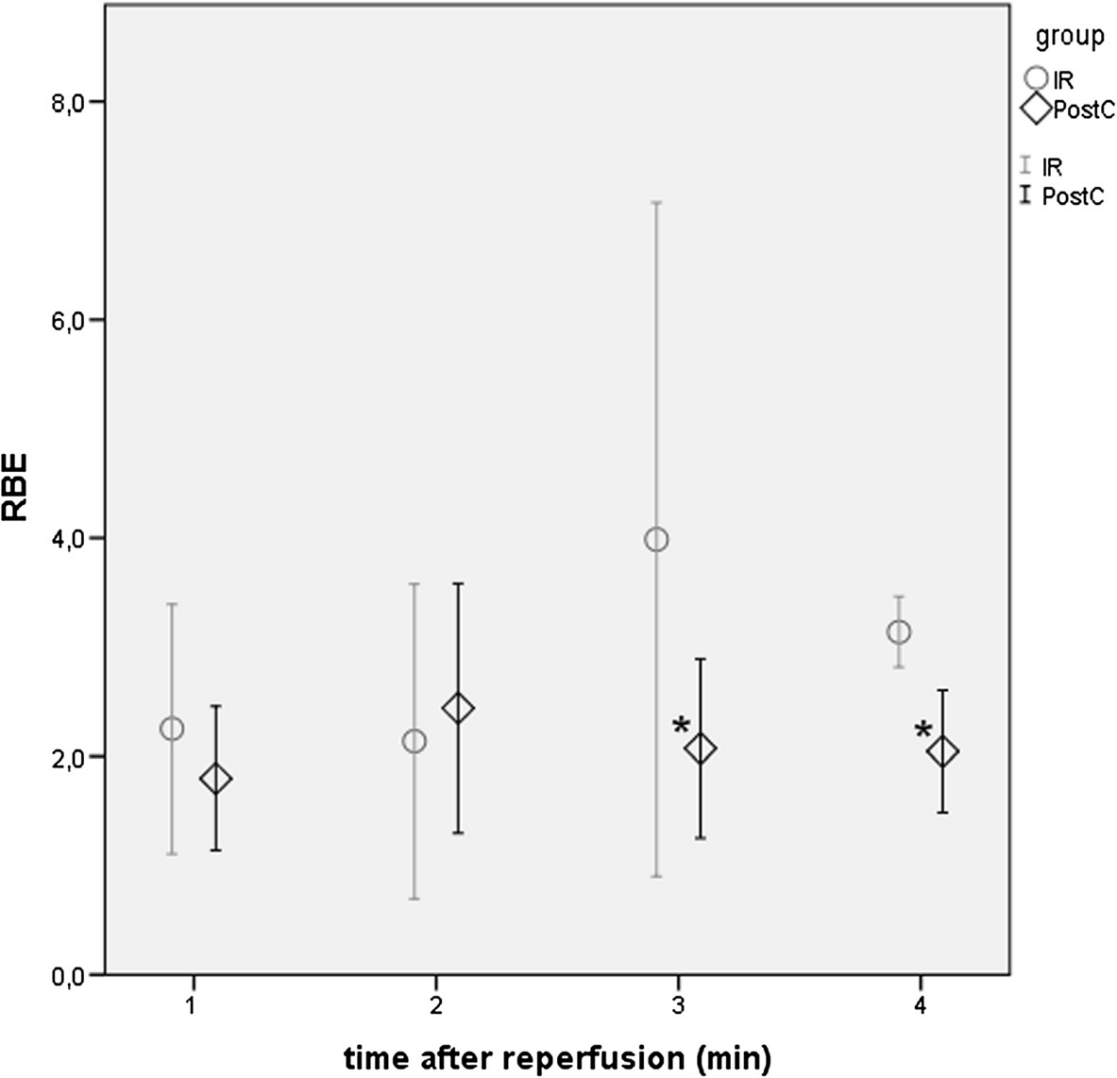
***Systemic acid***–***base status***: ***relative base excess*** ***(RBE).*** Base excess values are measured at the end of ischemia and at the 1^st^, 2^nd^, 3^rd^, 4^th^ min of reperfusion. Relative base excess (calculated as a quotient of the measured values and the base excess registered before reperfusion) is in direct relation with the systemic acidic load after lower limb muscle ischemia-reperfusion injury. In case of the postconditioned (PostC, ◊, n = 5) group the increase in RBE is lower compared to the ischemia-reperfusion (IR, ○, n = 5) group. Differences are statistically significant (*) at the 3^rd^ and 4^th^ min after the onset of reperfusion between the PostC and the IR animals.

Relative base excess (RBE) is an arbitrary parameter, formed as a quotient of each registered base excess values (BE, measured at 4 points of time at the beginning of the reperfusion) and the base excess value just before the onset of the reperfusion in the same animal. As a quotient of two negative numbers, a higher positive RBE represents a higher acid-load. PostC animals developed significantly less increased RBE values, than the IR animals (Figure 3).

### Hemodynamics

- (a) In the IR group, a higher ’shock-index’ occurred for the period of reperfusion as for ischemia (the quotient is >1), whereas in the group of the PostC animals the quotient was <1, showing a non-elevated shock-index after revascularization (IR: 1.04 ± 0.22 vs. PostC: 0.81 ± 0.19, p = 0.044).
- (b) The lowest systolic blood pressure within the first 20 min of reperfusion occurred at 9.41 ± 7.05 min in the PostC, and at 7.87 ± 5.06 min after revascularization in the IR group. This drop of blood pressure proved significantly lower in degree in the PostC group (IR: 17.11 ± 3.59% vs. PostC: 11.58 ± 6.04%, p = 0.038).
- (c) After the drop of the blood pressure, the pressure curve reached the plateau phase sooner in the PostC than in the IR group (IR: 22.92 ± 20.42 min vs. PostC: 13.90 ± 12.93 min after revascularization). Because of the high standard deviation, the difference was found non-significant (p = 0.565).

### Kidney microcirculation

**Figure 4:**
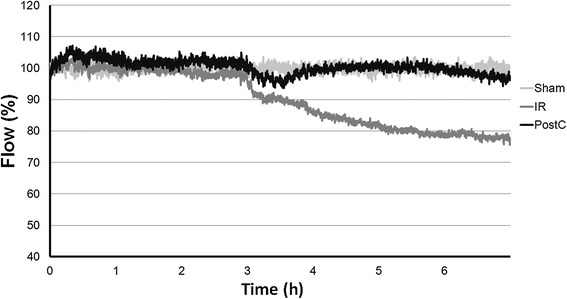
***Kidney cortex microcirculation measured with laser Doppler flowmeter*** **.** Measured flux is expressed as a percentage of the baseline flux before the aortic clamping. At revascularization flux dropped and for about 30 min remained at a lower, stable level in both IR (ischemia-reperfusion, n = 8) and PostC (postconditioned, n = 8) group. Thereafter microcirculation improved in the PostC group nearly reaching the normal baseline level, while it further deteriorated in the IR group. Shadowing marks the period of ischemia.

Kidney microcirculation remained at the baseline level after clamping of the infrarenal aorta. At revascularization flux dropped and after a lower, stable level, microcirculation improved in the PostC group nearly reaching the normal baseline level, while it further deteriorated in the IR group (Figure 4).

According to the calculated RA and PM, the perfusion of the kidney cortex was significantly better in the PostC group than in the IR group (RA: PostC: 99.01 ± 2.76% vs. IR: 82.31 ± 12.23%, p = 0.024, PM: PostC: 96.81 ± 6.14% vs. IR: 77.21 ± 14.81%, p = 0.037).

### HSP72 expression

**Figure 5:**
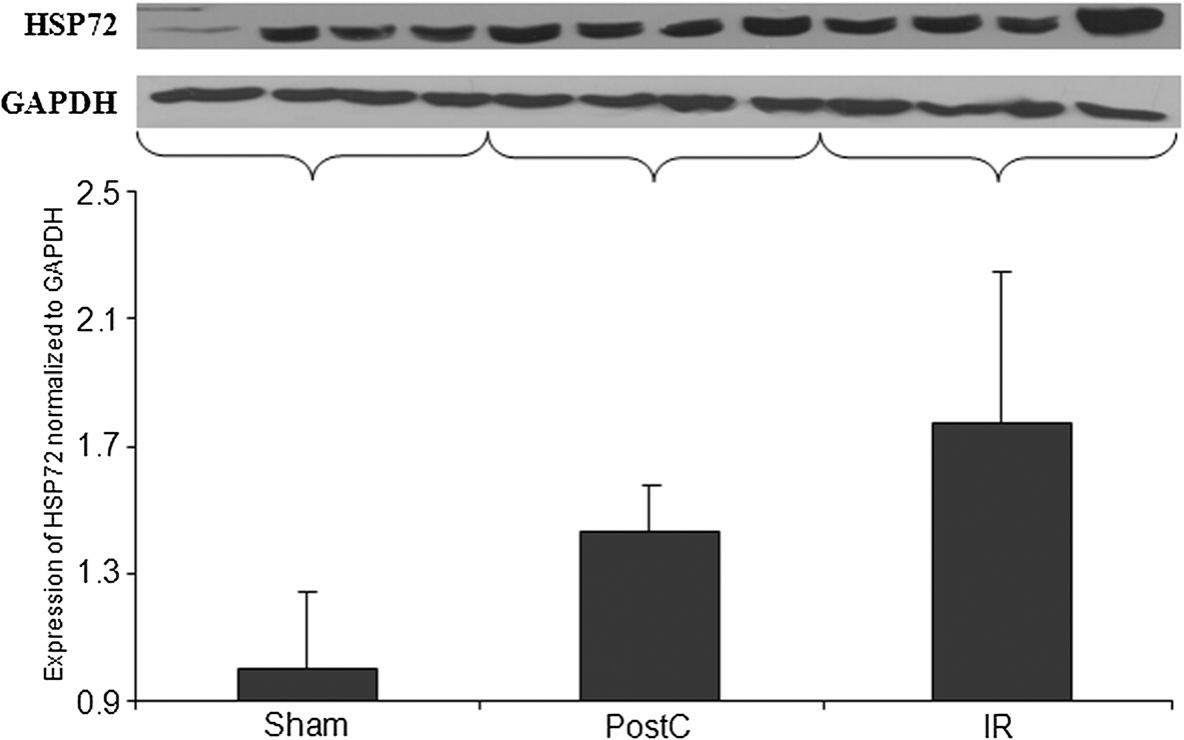
***Heat shock protein 72*** **(** ***HSP72*** **)** ***analysis*** **(** **HSP72 expression in the kidney samples** **).** Representative Western-blot bands are shown in the upper part of the figure. Analysis of kidney HSP72 expression normalized to glyceraldehyde 3-phosphate dehydrogenase (GAPDH) levels demonstrated no significant elevation in the IR (ischemia-reperfusion, n = 8) and PostC (postconditioned, n = 8) group compared to the sham-operated group (n = 5) after 4 h of reperfusion.

All HSP72 measurements were normalized and standardized by registering as a quotient of the housekeeping gene GAPDH expression in the same sample. Although there was a tendency for kidney HSP72 expression to be upregulated in both the IR and PostC groups vs. the sham-operated group, we did not find any significant differences between the groups (Figure 5).

### Lipid peroxidation

Conjugated diene contents detected by spectrophoto-meter from homogenized kidney samples were elevated in both IR and PostC animals in comparison with the sham-operated group, showing an early evolving lipid peroxidation in the post-revascularization 4^th^ hour. Conjugated diene concentrations (expressed in absorption units/ml) were significantly higher in the IR than in the PostC group (sham-operated: 0.12 ± 0.01, IR: 0.17 ± 0.04, PostC: 0.14 ± 0.01, p = 0.032).

## Discussion

One of the clinically most relevant and serious complications a vascular surgeon faces after aortic abdominal surgeries is a derangement in kidney function. In a small but not disregardable proportion of cases even acute kidney failure can be seen [13].

As being a still challenging task to solve, even requiring intensive care, we designed an animal model to further investigate renal dysfunction and kidney failure after major vascular surgery and also to find a method by which a vascular surgeon can take measures to prevent this serious complication.

Postconditioning has been deeply investigated in the last decade in different animal and human experimental models and has been proven to be a potent preventive method against myocardial IR injury [14]. A few animal studies have been reported in recent years about postconditioning and the attenuation of IR injuries also in other organs and tissues, including the kidney [15], the central nervous system [16], liver [17], lung [18] and the intestines [19]. The feature all the studies up to the present have in common is that they investigated only the local effects of postconditioning on IR injuries as well as the inflammatory changes after revascularization. The new concept and idea of our present study was to investigate not only the local protection of postconditioning, but also the effect it exerts upon other, remote, non-ischemic organs and on the systemic hemodynamics and metabolic balance.

Based on our previous experiments, we used a 3-hours limb ischemia model, with an ischemia long enough to represent a relatively long aortic reconstruction and to find clinical relevance by causing massive ischemic insult to the limb tissues and thus provoking high prevalance and degree of postoperative complications [9]. The first non-expected observation was in our model that no protective effect appeared on the early muscle IR injuries by using postconditioning, since previous studies reported otherwise [8,20]. A slightly lesser mitochondrial ischemic injury, or a higher or faster degree of mitochondrial regeneration, demonstrated by enzyme histochemical viability assessment was detected only 24 hours after reperfusion in the PostC animals. Studying the postoperative changes in renal function, a remarkable renal dysfunction was found in all animals subjected to IR. A significantly lesser increase in serum creatinine concentrations was registered in PostC group 4 hours after revascularization. Tracing the progression of kidney failure, calculated RFI demonstrated a propagation of dysfunction on the first postoperative day and a regression till the 72^nd^ postoperative hour. The difference between the subgroups still showed a lower degree of renal failure in the PostC animals.

The explanation of why we found no protective role of postconditioning for the early muscle injuries may remain disputable, or may be attributed to the model characteristics we used. Rosero et al. reported that significant collateral circulation exists in the lower limbs of rats originating from proximally of the infrarenal aorta [21]. A collateral vascular blood supply of the muscle fibres can presumably seriously effect the subcellular oxidoreductive events during ischemia-reperfusion and also during the application of additional reocclusion cycles. With use of other models (limb tourniquet with an absolute ischemia) we might have found perhaps other results in terms of the IR injury reducing effect of postconditioning, as stated in the study of Wang et al. [22]. In any case, the remarkable observation of no positive effect on the early reperfusion injuries, but an apparent, significant protective role against the renal dysfunction must underline some other mechanisms, the interaction of postconditioning with kidney injuries, which have not been revealed yet.

Ischemic rhabdomyolysis during vascular reconstructions provokes ’renal’ (parenchymal) type of acute renal failure, due to multifactorial mechanisms of myoglobin toxicity on the renal parenchyma [23]. After released from the injured myocytes, myoglobin, being a small molecule (16.7 kDa), easily escapes in the urine, where it interacts with Tamm-Horsfall proteins resulting in tubular obstruction [24]. Furthermore, myoglobin-degradation and metabolism in (especially the proximal) tubular cells leads to free-radical mediated heme toxicity, redox-imbalance and lipid peroxidation, as well as a reduction of the kidney microcirculation by the ability of myoglobin to scavenge nitric oxide and to participate in redox cycling of other vasoactive mediators [25,26]. Intraluminal precipitation and all mechanisms of myoglobin-mediated renal damage are augmented at acidic pH of the urine [27,28].

In this present investigation, myoglobin toxicity seemed less expressed on anti-myoglobin stained kidney samples, which was confirmed by the results of diene conjugate measurements: the PostC group showed significantly lesser oxidative inury. At the same time, possibly due to a well-compensated metabolic balance, systemic blood pH did not alter from the baseline level measured at the end of ischemia, but a fall could be observed in the base excess values, which decrease was significantly less pronounced in the PostC animals.

Besides the myoblobin-related acute tubular damage, ’prerenal’ components of azotemia also develop [29]. Revascularization of a previously ischemic, dilated microvasculature with a compromise in autoregulation leads to serious systemic hemodynamic disturbances. A fluid shift can occur into the damaged muscle tissues from the intravascular compartment and a relative hypovolemia results [9]. Renal blood flow rapidly decreases and prerenal azotemia is an appropriate, physiological response to renal hypoperfusion and the neuroendocrine stress reaction [30]. We performed a continuous registration and a detailed analysis of systemic hemodynamic parameters, and the results revealed a significantly better compensated circulatory impairment in the PostC animals. Parallel with the systemic changes, renal microcirculation measured with laser Doppler flowmeter also showed a significantly lesser degree of hemodynamic derangement on the level of the microvessels.

Heat shock proteins have a significant role in the protection against IR injury [31]. Levels of HSP72 have been demonstrated to be elevated in remote organs (including the kidney) after limb IR injury [32]. Our result also showed elevation of HSP72 in the kidney samples, however there was no significant difference between the groups. This suggests that the renal protecting effect of lower limb postconditioning is independent of changes in HSP72 expression in the early stages of injury.

Laboratory measurements and calculated renal functional parameters revealed a kidney failure of mainly tubular type, emerging early after reperfusion in the IR group, while these parameters remain this time closer to the normal values in the PostC group. One and three days after revascularization, a much more pronounced ’renal type’ of kidney failure is present in both subgroups, but still a significant difference can be observed between the PostC and the non-conditioned (IR) animals. In our explanation, the fact of an essentially tubular type of failure does not necessarily exclude a possible, lesser degree prerenal component of azotemia to exist at the same time, which can be demonstrated by our findings about the systemic and the kidney cortex circulatory conditions.

Furthermore, this study raises new hypotheses about the protective potential postconditioning possesses. The fact of no positive effect in this animal model on the lower limb IR injuries but the substantial protection the method conferred against the resultant renal dysfunction gives proof for new theories about postconditioning. Altering the release of toxic metabolites, myoglobin and acidic substances from the injured limb tissues makes a prolonged but thus lesser degree of acidic and toxic load in the systemic circulation and urinary filtrate, while it impacts a milder disturbance of systemic hemodynamics, affecting autoregulatory mechanisms on systemic and local microcirculatory levels as well.

Our results and experimental observations present convincing evidence about the positive impact of postconditioning on renal complications after ischemic rhabdomyolysis and make a reasonable promise for a possible future application in different clinical situations. Newer studies drew attention to certain concerns about the actual effectiveness of the method under circumstances with comorbidities of diabetes, hypertension, hypercholesterolemia (conditions that often affects patients with arterial occlusive disease), which fact could mean some limitations of the current model [33,34]. However, our findings about the effectiveness of postconditioning in terms of renal protection that seemed (at least partially) independent of the effect (or inefficiency) on the muscle IR injuries suggest the possibility of a successful translation into clinical practice. The easy and practicle attainability of the method and the positive impact on systemic hemodynamics during the operations will hopefully lead us to take measures to prevention of postoperative kidney disfunction.

## Conclusions

In this study we set up a rat model of bilateral lower limb ischemia-reperfusion injury to represent a model of major lower extremity arterial reconstructive operations in which muscle IR injuries and the consequential postoperative complications, the evolving renal dysfunction can be studied. With application of the method postconditioning a significantly lesser degree of renal functional impairment was detected, though this renal protection seemed independent of the limb muscle injuries, where postconditioning resulted in no significant effect in the early postrevascularization period. We aimed at searching for explanations for these findings by investigating different aspects and the underlying mechanisms of the evolving kidney dysfunction. We found that a dominantly parenchymal type of kidney failure was characterized by myoglobin toxicity on the tubular cells, and systemic acidic load, postconditioned animals expressed less severe myoglobin-induced tubular injury and a better compensated acid–base status. On the other side, our results are suggestive of a multifactorial mechanism of acute kidney failure: postconditioning also had a positive impact on the microcirculation of the kidney cortex and the systemic hemodynamic parameters, indicating a lesser degree of prerenal azotemia.

The study reveals some new aspects of postconditioning, its mechanical effect on hemodynamic stability and the gradual release of toxic substances from the injured tissues, resulting in kidney protection after major vascular operations.

## Abbreviations

ANOVA: Analysis of variance
BE: Base excess
DBP: Diastolic blood pressure
DTT: Dithiothreitol
FENa: Fractional sodium excretion
GAPDH: Glyceraldehyde 3-phosphate dehydrogenase
HEPES: 4-(2-hydroxyethyl)-1-piperazineethanesulfonic acid
HR: Heart rate
HSP72: Heat shock protein 72
IR: Ischemia-reperfusion
KCl: potassium chloride
MAP: Mean arterial pressure
MgCl_2_: Magnesium chloride
Na: Sodium
NADH: Nicotinamide-adenine-dinucleotide
NBT: Nitroblue tetrazolium chloride
PostC: Postconditioned
PM: Plateau maximum
PMSF: Phenylmethylsulfonyl fluoride
RA: Reperfusion area
RBE: Relative base excess
RFI: Renal failure index
RGB: Red-green-blue
SBP: Systolic blood pressure
SD: Standard deviation
TRIS: Tri-hydroxymethyl-aminomethane
